# The role of candidate transport proteins in β‐cell long‐chain fatty acid uptake: Where are we now?

**DOI:** 10.1111/dme.15198

**Published:** 2023-09-02

**Authors:** Christina Clavelo‐Farrow, Patricia Thomas

**Affiliations:** ^1^ Institute of Metabolism and Systems Research, University of Birmingham Birmingham UK

**Keywords:** β‐cells, candidate transport proteins, fatty acid uptake, lipotoxicity, long‐chain fatty acids

## Abstract

Type 2 diabetes (T2D) in humans is typically preceded by elevated levels of circulatory long‐chain free fatty acids (LC‐FFA). These excess LC‐FFA are widely thought to be taken up by pancreatic β‐cells, contributing to their dysfunction and death during the development of T2D; a process that has been termed lipotoxicity. Depending on their degree of saturation and carbon chain length, LC‐FFA can exert different effects on pancreatic β‐cells viability and function in vitro. Long‐chain saturated fatty acids (LC‐SFA) are thought to be toxic, whereas monounsaturated fatty acids are not and may even offer protection against the toxic effects of LC‐SFAs. However, the mechanism of LC‐FFA uptake into pancreatic β‐cells is poorly understood, partly because it has been an understudied area of research. Determining how LC‐FFA are taken up into β‐cells is crucial for later formulation of therapies to prevent potential cellular overload of LC‐FFA, thereby slowing the onset of T2D. In this work, we detail more than 40 years of literature investigating the role of membrane‐associated transport proteins in LC‐FFA uptake. By focussing on what is known in other cell types, we highlight where we can extrapolate our current understanding of protein‐mediated transport to β‐cells and uncover where further understanding is required.

**Key Points**

- Many individuals with type 2 diabetes (T2D) have elevated circulatory concentrations of long‐chain saturated fatty acids (LC‐SFA) which are widely believed to contribute towards pancreatic β‐cell dysfunction in the development of T2D.
- Currently, it remains unknown how LC‐SFA cross the plasma membrane of β‐cells (or indeed any cell type).
- Here, we describe what is currently known about the potential mechanisms of membrane‐associated LC‐SFA transport proteins to better understand LC‐SFA uptake in β‐cells.
- Understanding the mechanisms of LC‐SFA uptake in β‐cells would enable therapeutics to be formulated which regulate their entry for the treatment of T2D.

## 1 INTRODUCTION

The mechanism of long‐chain fatty acid uptake (LC‐FFA) into insulin‐producing pancreatic β‐cells is poorly understood. Fatty acids (FFA) are a heterogeneous group of compounds used by the cell to facilitate function, including as an energy source, as a constituent of the cell membrane, and, in β‐cells, to potentiate glucose‐stimulated insulin secretion (GSIS).^1^ In excess, they are believed to cause β‐cell dysfunction and death during the development of type 2 diabetes (T2D) in a process that has been termed ‘lipotoxicity’. At present, it remains unclear how LC‐FFA cross the plasma membrane of β‐cells (or any cell type^2^). Identifying the underpinning mechanism of LC‐FFA uptake would enable therapies to be developed which regulate their entry into β‐cells, thereby reducing the toxic effects of LC‐FFA and potentially slowing the progression of T2D.

In this review, we probe the current understanding of candidate LC‐FFA transport proteins to gain an overview of the molecular mechanisms underpinning uptake. Over the past 40 years, many studies have been conducted with the aim to identify those proteins that serve to transport LC‐FFA across the plasma membrane.^2^ However, as uncovered in this review, few of these studies pertain to β‐cells. Throughout this manuscript, we discuss how the current understanding of protein‐mediated LC‐FFA uptake in other cell types can be extrapolated to pancreatic β‐cells and highlight key questions that should be considered in future studies.

## 2 THEORIES OF LONG‐CHAIN FATTY ACID UPTAKE

The mechanism by which LC‐FFA translocate the plasma membrane of cells has been extensively debated for many years (as discussed by Glatz and Luiken^2^), with the central question being whether the process occurs via passive diffusion or is protein‐mediated. Historically, it was believed that LC‐FFA enter cells only through passive diffusion, largely due to their amphipathic nature.^2^ The physical basis for this theory lies in the fact that the hydrophobic core of the plasma membrane acts as a barrier to hydrophilic molecules. The polar headgroup and nonpolar chain of LC‐FFA provide the necessary biophysical properties to permeate the phospholipid bilayer of the membrane using a proposed three‐step ‘flip‐flop’ mechanism.^3^ LC‐FFA are thought to be released from the albumin carrier, and the hydrocarbon chain then intercalates between the phospholipid chain of the membrane, with its carboxyl group localising to the aqueous interface (adsorption). The LC‐FFA then moves through the phospholipid bilayer (‘flip‐flop’ or translocation) and dissociates from the membrane (desorption).^2^ It is believed that desorption is the rate‐limiting step of LC‐FFA diffusion.^2^, ^4^ Passive diffusion as the mechanism for LC‐FFA uptake is supported by studies using artificial phospholipid bilayer vesicles and mathematical models which show that LC‐FFA can rapidly diffuse across the membrane in the absence of proteins.^4^, ^5^

**FIGURE 1:**
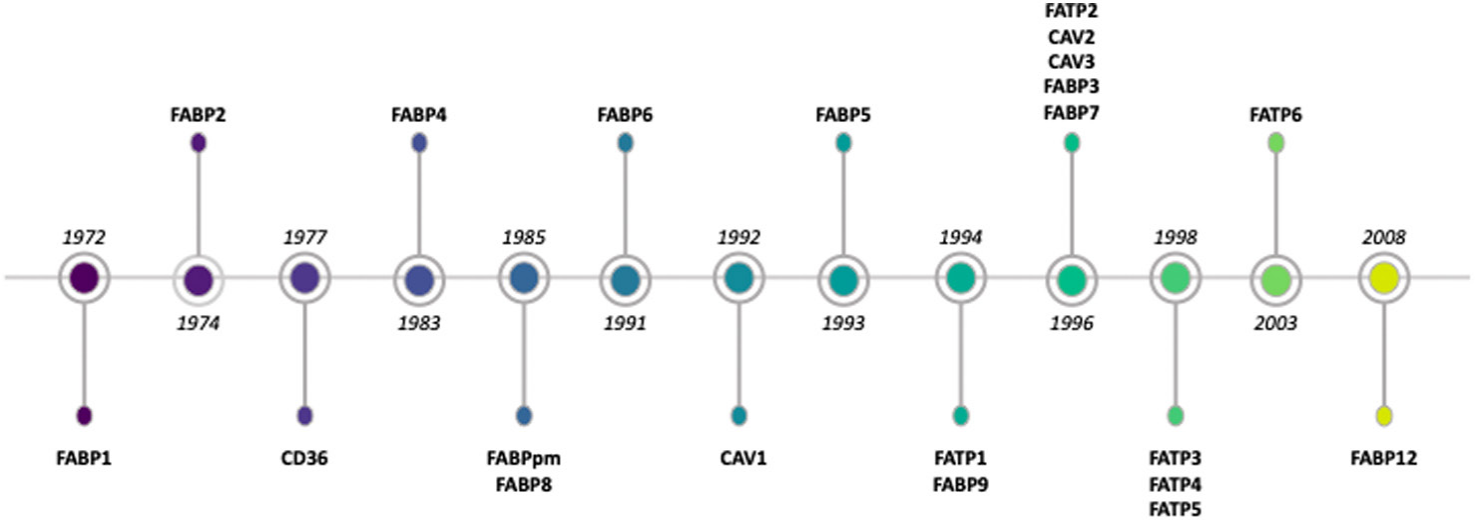
Timeline of the discovery of candidate LC‐FFA transport proteins identified within this review. CAV: caveolin; FABP: fatty acid binding protein; FATP: fatty acid transport protein.

As discussed by Glatz and Luiken,^2^ the search for candidate LC‐FFA transport proteins was first triggered by the suggestions that: (a) it would be physiologically undesirable to have LC‐FFA enter cells unregulated; and (b) as LC‐FFA are a major fuel source for cells, their uptake can be analogised to glucose which requires, and is regulated, by glucose transporters. Under this theory, transport proteins would therefore provide a means of controlling the rate at which LC‐FFA enter cells. Since the early 1970's, several candidate LC‐FFA transport proteins have been identified (Figure 1). Loss‐ and gain‐of‐function studies (Table S1) support that candidate transport proteins may play a role in LC‐FFA uptake in a wide range of cell types. However, studies are conflicting as to which transport protein/family of transport proteins mediate uptake. In β‐cells, LC‐FFA uptake is often attributed to the scavenger receptor, CD36,^6^ although, as discussed later in this review, there is little evidence to support this in this cell type.

FFA are particularly important to β‐cells as they augment GSIS^1^ and may even induce insulin secretion at fasting glucose concentrations.^7^ The mechanism by which this occurs is not fully understood but it is known to be partially mediated by the activation of the cell membrane G protein‐coupled receptor (GPCR), free fatty acid receptor 1 (FFAR1) (previously GPR40).^8^ FFA‐FFAR1 signalling can potentiate GSIS by mobilising endoplasmic reticulum (ER) calcium stores and promoting filamentous actin remodelling for greater access of insulin secretory granules to the plasma membrane (for a more complete understanding see Campbell & Newgard^9^).

FFA uptake serves as the initial step to regulating GSIS through intracellular metabolic pathways.^8^ The metabolism of exogenous (e.g. FFA in circulation) and endogenous FFA (e.g. lipolysis of lipid droplets and/or membrane lipids) generates metabolic coupling factors which are thought to further contribute towards insulin secretion.^8^ For example, the glycerolipid/FFA cycle has emerged as a potential pathway that couple's lipid and glucose metabolism in GSIS.^9^ Briefly, glucose entering the β‐cell causes an increase in malonyl CoA, which promotes FFA esterification.^9^ Through a series of sequential reactions, glycerol‐3‐phosphate (generated from glucose) has two FFA chains attached to form phosphatidic acids.^8^ Phosphate of phosphatidic acids is removed by lipin to generate 1,2‐diacylglycerol which then undergoes further reactions to form triacylglycerol which is stored in lipid droplets.^8^ Through a process of lipolysis, FFA are released from their intracellular stores and reused to form triacylglycerol or secreted to act on cell surface membrane receptors such as FFAR1.^8^ Metabolites generated through the glycerolipid/FFA cycle are thought to increase GSIS (for a more complete overview see Imai et al.^8^). It could therefore be argued that it is physiologically advantageous to regulate the concentration of FFA entering the β‐cell, a process that would require transport proteins. As healthy β‐cells possess a means to store and release FFA in times of excess fuel^10^ passive diffusion may still play a role; within this review, however, we assess the evidence for candidate transport proteins.

## 3 CANDIDATE LONG‐CHAIN FATTY ACID TRANSPORT PROTEINS

Over the past 40 years, many proteins have been proposed to play a role in mediating LC‐FFA uptake, often with conflicting results. In our analysis of the literature (Table S1), we identified 21 candidate transport proteins that function individually or in concert to facilitate LC‐FFA uptake. Across cell types, fatty acid translocase (FAT)/CD36 (hereafter, referred to as CD36) has the most literature to support a role in mediating LC‐FFA uptake, followed by fatty acid transport proteins (FATP) (Table S1).

### 3.1 CD36‐mediated LC‐FFA uptake

**FIGURE 2:**
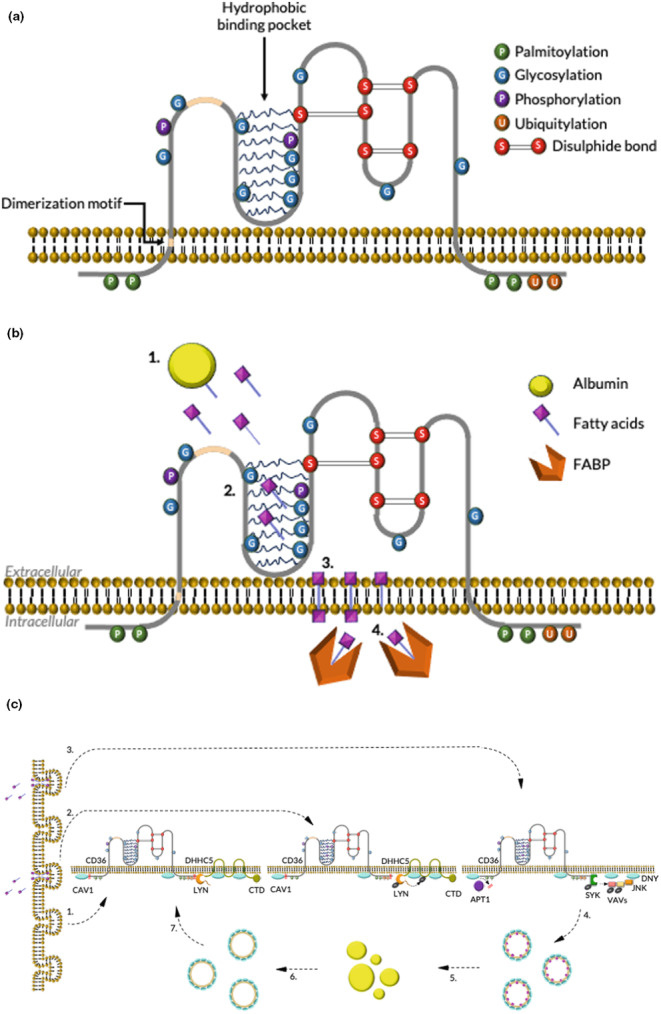
CD36 and its proposed mechanism of fatty acid uptake. (a) Structure of CD36 with post‐translational modifications (adapted from Moon et al.^9^). (b) Uptake of FFA facilitated by CD36 and fatty acid binding protein (FABP) (adapted from Glatz and Luiken^2^). (1) FFA dissociate from albumin. (2) FFA bind to the hydrophobic cavity of CD36. (3, 4) FFA is guided through CD36 to the outer leaflet of the membrane where it adsorbs into the plasma membrane. (5) FFA translocation. (6, 7) desorption from the inner leaflet and binding to FABP which is anchored to the intracellular domain of CD36. (c) CD36‐mediated endocytosis (adapted from Hao et al.^10^). (1) within caveolae‐enriched membranes, the caveolae contain CD36 in its palmitoylated form, with CAV1 on its inner layer. (2) FFA binds to CD36 activating the SRC kinase, LYN, which phosphorylates and subsequently deactivates DHHC5. (3) Deactivation of DHHC5 leads to the depalmitoylation of CD36 by APT1. Depalmitoylated CD36 recruits SYK which phosphorylates JNK and VAV leading to (4) CD36‐mediated caveolar endocytosis. (5) Endocytic vesicles are then delivered to lipid droplets to deliver their FFA cargo for storage. (6) CD36 is repalmitoylated and (7) recycled to the plasma membrane.

Multiple studies have shown that the overexpression or inhibition of CD36 in metabolically active cell types (Table S1) alters the rate of LC‐FFA uptake. CD36 is a transmembrane receptor (Figure 2a) which is anchored to the plasma membrane through palmitoylated cysteine residues on its N‐ and C‐terminal tails.^11^ Up to two FFA are thought to bind to the hydrophobic cavity of CD36, with palmitoylation of CD36 being well documented to play a role in the regulation of uptake.^11^, ^12^ CD36 has a range of functions in β‐cells, for which we refer the reader to the review of Moon and colleagues.^11^ Here, however, we discuss only its role in FFA uptake.

CD36 is present in varying islet cell types including β‐ and alpha cells. In β‐cells, it localises to the plasma membrane and insulin secretory granules.^13^ CD36 mediates lipid accumulation^14^ but in the published literature, it is widely attributed to facilitating β‐cell LC‐FFA uptake^6^ but has not been extensively studied. There are seemingly only two studies^13^, ^15^ that have shown CD36 to play a role in LC‐FFA uptake in β‐cells. By inhibiting CD36 with the LC‐FFA, sulfosuccinimidyl‐oleate, Noushmehr et al.^13^ reported a 46 ± 9.7% inhibition of palmitate uptake in the mouse‐derived MIN6 cell line, compared to vehicle control. Through the forced overexpression of CD36 by doxycycline, Wallin et al.^15^ observed a 41 ± 4% increase in uptake of the long‐chain monounsaturated fatty acid (LC‐MUFA) oleate. Together, these studies support that CD36 may play a role in LC‐FFA uptake in β‐cells.

A review of the literature yields many potential mechanisms for how CD36 facilitates FFA uptake. We present two of these mechanisms in Figure 2b,c. During the pathogenesis of T2D, it has been suggested that CD36 plays a pivotal role in β‐cell glucotoxicity; the dysfunction of β‐cells through chronic exposure to supraphysiological concentrations of glucose.^15^ Elumalai et al.^16^ theorise that under conditions of high glucose, the small GTPase, Rac1, activates NADPH oxidase, inducing CD36 trafficking to the plasma membrane. Elevated CD36 plasma membrane expression increases FFA uptake which downstream leads to enhanced reactive oxygen species (ROS) formation and β‐cell apoptosis through mitochondrial dysfunction.^16^ However, this pathway requires further characterisation as CD36 is related to a diverse number of cellular processes, and the direct interaction between NADPH oxidase and CD36 is yet to be demonstrated.^11^ In summary, the literature supports that CD36 facilitate β‐cell LC‐FFA uptake, although further research is needed. Characterising the mechanism of CD36‐mediated uptake would provide insight into understanding what role this protein plays in β‐cell failure during the development of T2D.

### 3.2 FATP‐mediated LC‐FFA uptake

**FIGURE 3:**
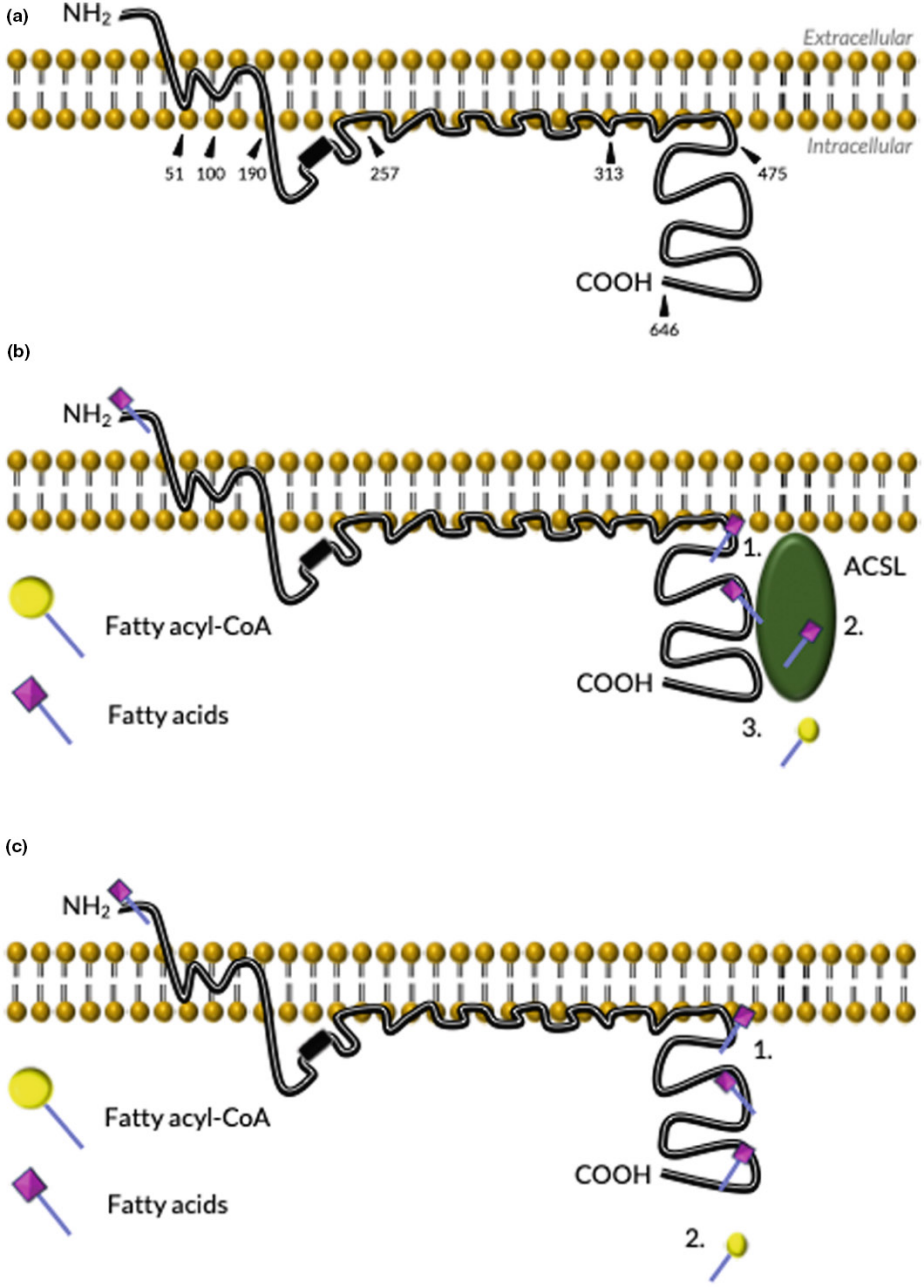
FATP and its proposed mechanisms of LC‐FFA uptake. (a) Proposed topology of FATP1 (adapted from Lewis et al.^26^). Numbers refer to amino acid residues, with 246–254 being the AMP‐binding motif, and 246–557 the FATP signature. FATP4 contains an ER localisation domain at amino acid residues 47–102. (b) Membrane‐bound FATP and long‐chain acyl‐coenzyme A synthases (ACSL) function co‐operatively to transport and activate LC‐FFA. (c) Membrane‐bound FATP transport and activates LC‐FFA (adapted from Arias‐Barrau et al.^27^).

The FATP (also known as SLC27) family are integral membrane proteins (Figure 3a) consisting of six isoforms, FATP1‐6, whose expression is tissue‐specific.^17^ Numerous studies support that FATPs mediate FFA uptake across cell types, with specificity for LC‐FFA and/or very‐long chain FFA.^18^ With an AMP and fatty acid binding domain, FATP proteins have a domain architecture similar to acyl‐CoA synthetases which catalyse FFA thioesterification, a process that ‘activates’ the FFA for downstream β‐oxidation and the synthesis of TAG and complex lipids.^18^ Whether FATP act to (a) transport fatty acids across the plasma membrane, (b) as an acyl‐CoA synthetase, metabolically trapping FFA by a process termed vectorial acylation or (c) form a complex with an acyl‐CoA synthetase isoform to catalyse transport (Figure 3b‐c)^19^ is widely studied.

Little is known regarding FATP function and expression in β‐cells. FATP2 is up‐regulated in human islets following high‐glucose stimulation^20^ and has been shown to be the predominant isoform in the rat‐derived, INS‐1E, β‐cell line.^21^ The up‐regulation with high glucose exposure^20^ suggests that β‐cells may increase their FFA uptake via FATP2 in times when GSIS amplification is necessary. However, further work is required to support a mechanistic basis of FATP2 in β‐cell LC‐FFA uptake. FATP2 mediates FFA uptake in other cell types^22^ but has also been shown to play a role in lipid metabolism (as discussed by Anderson and Stahl^17^), most notably as a contributor to peroxisomal very long‐chain acyl‐CoA synthetase activity in hepatocytes.^22^ Future studies in β‐cells should consider both splice variants, FATP2a and FATP2b. FATP2 acts as both an FFA transporter and acyl‐CoA synthetase, while FATP2a has only acyl‐CoA synthetase activity with a preference for very‐long‐chain FFA.^18^ Characterising the subcellular localisation and role of both splice variants in β‐cells may elucidate whether FATP2 (a) mediates uptake, (b) acts as a transporter and/or through its acyl‐CoA synthetase activity to mediate LC‐FFA uptake, (c) has FFA specificity.

FATP1 is highly expressed in skeletal muscle, heart and adipocytes, with lower levels of expression in the liver, lung, brain and kidney.^17^ Insulin has been shown to be critical for FATP1 to facilitate LC‐FFA uptake.^17^ Multiple studies^23^, ^24^ have shown insulin to induce FATP1 translocation from intracellular depots (e.g. the perinuclear compartment) to the plasma membrane, leading to increased LC‐FFA uptake in muscle and adipose. If FATP1 does require insulin to facilitate uptake in β‐cells, this could imply that LC‐FFA uptake occurs through the autocrine action of insulin. As FFA potentiates GSIS, the theory of FATP1 as a candidate transport protein for LC‐FFA uptake in β‐cells aligns with the theory of autocrine control of insulin secretion^25^; a concept which remains controversial and requires substantial investigation in human β‐cells.

Similarly to FATP1, FATP4 is expressed in a wide range of tissue. However, unlike FATP1, FATP4 is present in both insulin‐sensitive and insulin‐insensitive tissue.^18^ FATP4 has been found to localise to the ER and drive FFA uptake indirectly via acyl‐CoA synthetase‐mediated activity,^28^ with substrate specificity for palmitate and lignocerate.^29^ Insulin has been shown to increase the activity of FATP4. Digel et al.^30^ observed that in muscle cells, FATP4 overexpression causes localisation to the ER and an increase in acyl‐CoA synthetase activity coupled with oleate uptake. Both effects were sensitive to the inhibition and treatment of insulin.^29^ Zhan and colleagues^31^ propose that both FATP1 and FATP4 localise to the ER in adipocytes, with insulin increasing FFA uptake without plasma membrane translocation. Hamilton and Brunaldi^32^ suggest that in endothelial cells of the brain, it is FABPs (FABP5) that traffic FFA from the plasma membrane to the ER‐localised FATP4. Alternatively, a stimulus such as insulin may increase the activity of FATP4, increasing the formation of acyl‐CoA and resulting in an inward FFA gradient in the cell.^33^ As FATP4 is present in both insulin and non‐insulin specific tissue it should be considered a candidate LC‐FFA transport protein in β‐cells and thus investigated further. If, like other tissues, it resides at the ER, then proteins that traffic FFA to FATP4 should also be characterised as potential therapeutic targets.

Peroxisome proliferator‐activated receptors (PPARs) are an FFA‐activated family of nuclear receptors, with the biochemical and expression profiles of the three subtypes being tissue dependent. Strikingly, PPAR⍺ and PPARβ/𝛿 have been shown to be protective against β‐cell lipotoxicity.^34^, ^35^ The *Slc27a1* promoter contains a PPAR response element which can bind and up‐regulate FATP1 expression.^36^ PPAR⍺ and PPARγ regulate FATP4 in adipocytes and hepatocytes^18^ with pharmacological activators increasing oleate uptake.^36^, ^37^ As FFA activates the transcriptional activities of PPARs, a positive feedback cycle may occur whereby FFA enter the β‐cell via an alternate mechanism, leading to the up‐regulation of FATP1 and/or FATP4 and increased FFA uptake.

It is unlikely that FATP3, FATP5 and FATP6 play a role in β‐cell LC‐FFA uptake. Doubts have been raised regarding the role of FATP3 in FFA uptake,^38^ and FATP5 and FATP6 are reported mostly in the liver and heart, respectively.^18^ In summary, FATP2 has the most evidence as a candidate transport protein for LC‐FFA uptake in β‐cells. Due to their broad distribution and activity as FFA transporters, the role of FATP1 and FATP4 in β‐cells merits further investigation.

## 4 FUTURE STUDIES IN β‐CELLS

Research into the role of candidate transport proteins in LC‐FFA uptake has mostly focussed on studies in adipocytes (Table S1); there are few studies on β‐cells. As the processing and utilisation of LC‐FFA are often unique to the cell type it must be taken into consideration that the mechanism of LC‐FFA uptake may differ in β‐cells compared to other, well‐characterised, tissues. The function of the individual candidate LC‐FFA transport protein may also differ between cell types. In a family carrying a Pro90Ser CD36 mutation, homozygotes but not heterozygotes had a reduced palmitate uptake in muscle and adipose tissue under palmitate‐suppressed conditions.^39^ Conversely, hepatic palmitate uptake was the same in those individuals with the homozygous and heterozygous mutation and matched controls.^39^ This suggests that the role of CD36 is cell‐type specific, thus the function of candidate LC‐FFA transport proteins should be considered in β‐cells and not presumed from other cell types. However, as CD36 and certain FATP family members have been shown to facilitate LC‐FFA uptake across a wide range of cell types (Table S1), their roles in β‐cells merit further investigation.

The role of CD36 and FATPs in LC‐FFA uptake has been demonstrated in varying human and rodent cell types (Table S1). Emerging evidence has shown interspecies differences in lipid handling in rodent vs. human β‐cells^40^ and there are marked differences in the expression of the principal glucose transporter (GLUT1 instead of GLUT2).^41^ In human, but not mouse, placental trophoblasts, *FATP2* and *FATP4* expression are regulated during hypoxic stress, a condition that has been associated with reduced fetal fat supply.^42^ Whether there is a species‐specific difference in isoforms, patterns of expression, or the relative abundance, etc., of the β‐cell candidate LC‐FFA transporters should be considered in future studies.

Most research into protein‐mediated LC‐FFA uptake measures the rate of palmitate and oleate (Table S1) transport across the membrane. The plasma FFA profile comprises >30 FFA species, with palmitate, oleate and stearate making up 78% of total FFA in circulation.^43^ FFA are a diverse species due to their combination of carbon chain length, and degree and location of unsaturation (and thus conformation). Due to FFA diversity, candidate transport proteins may have specificity and/or affinity for different FFA species. This is demonstrated by FFA‐activated GPCRs, whereby FFAR1 and FFAR4 (previously GPR120) are activated by medium and long‐chain FFA, whereas FFAR2 (previously GPR43) and FFAR3 (previously GPR41) are activated by short‐chain FFA.^44^ As such, a greater range of FFA should be used in future studies investigating the mechanism of LC‐FFA uptake in β‐cells. This would account for the potential specificity and/or affinity of the candidate transport protein to different LC‐FFA species and is more reflective of the situation in vivo, where β‐cells are exposed to a range of LC‐FFA in circulation.

The concentration of LC‐FFA that the β‐cell transporter is exposed to is also of importance. In the study conducted by Hames,^39^ homozygotes for a Pro90Ser CD36 mutation, but not heterozygotes have a reduced palmitate uptake in muscle and adipose tissue under palmitate‐suppressed conditions.^39^ When palmitate concentrations are moderately increased, homozygotes for the Pro90Ser CD36 mutation have no difference in the rate of palmitate uptake in muscle and adipose relative to matched controls.^39^ Carley and Kleinfeld^45^ also found that CD36‐facilitated oleate uptake is greater in cardiomyocytes at lower concentrations and declines as the concentration increases. This suggests that CD36 can only facilitate uptake at low FFA concentrations potentially through its saturation. This is not universally observed, however, as Lynes et al.^46^ report that in mouse enterocytes a high‐fat diet is required for the up‐regulation of CD36 to enhance CD36‐dependent FFA uptake. In future studies of β‐cells, a range of FFA concentrations should be used to account for the potential saturation and/or up‐regulation of the candidate LC‐FFA transporter.

## 5 INHIBITING LC‐FFA UPTAKE AS A THERAPEUTIC STRATEGY FOR T2D

By identifying the mechanism underpinning LC‐FFA uptake in β‐cells, new drug targets may be revealed that regulate LC‐FFA entry. Emerging studies suggest that blocking FFA uptake using FATP2 and CD36 inhibitors has therapeutic potential for T2D. The FATP2 inhibitors, Lipofermata (CB16.2) and Grassofermata (CB5) prevent FFA uptake and protect against LC‐SFA‐induced cell death in a range of cell types in vitro, including β‐cells.^21^, ^47^ When tested in vivo, both inhibitors prevent FFA uptake at the intestinal epithelium in mice dosed orally.^21^, ^47^ Small molecular weight compounds (AP5055 and AP5258) with anti‐CD36 activity have also been shown to prevent FFA uptake and protect against atherosclerotic plaque growth, improve glucose tolerance and reduce postprandial hyperlipidaemia in rodent models.^48^ Interestingly, Moon et al.^49^ propose that the antihyperglycaemic agent, metformin, may protect β‐cells against glucotoxicity, in part, by inhibiting CD36‐mediated FFA influx. These studies provide proof of concept that regulating FFA uptake is a conceivable treatment for T2D, but the potential for side effects cannot be overlooked.

In individuals with T2D, enhanced FFA uptake may be a compensatory response to a reduction in energy supply from glucose. Umbarawan et al.^50^ propose that for such individuals, limiting FFA uptake may be detrimental to cardiac contractile dysfunction as it would limit the heart's fuel supply. Conversely, Angin et al.^51^ showed that blocking CD36‐mediated palmitate uptake prevents lipid‐induced contractile dysfunction in cardiomyocytes. This raises the question, would inhibiting LC‐FFA uptake limit fuel supply to β‐cells, thus inducing dysfunction, or protect against nutrient overload thereby preventing dysfunction? Moreover, inhibiting CD36 can impair the removal of dead cells by macrophages which can lead to adverse left ventricular remodelling and post‐myocardial infarction.^52^ Thus, inhibiting candidate LC‐FFA uptake proteins, such as CD36, may have other implications than just inhibiting FFA uptake. If cellular FFA uptake is inhibited locally it poses the question of whether FFA would remain in circulation, increasing the risk of vascular disease in those with T2D; robust compound testing is required. Gaining a clear understanding of the mechanism of LC‐FFA uptake in β‐cells will increase the likelihood of drug success and the ability of monitoring/mitigating for potential side effects. Overall, however, the work currently being conducted on FFA uptake inhibitors in rodents suggests that regulating uptake may be a potential treatment for T2D.

## 6 CONCLUSION

CD36 and FATP1, FATP2 and FATP4 have the most evidence to support a role in palmitate and oleate uptake. However, research into the function of CD36 and FATP isoforms as LC‐FFA transporters has mostly been investigated in adipocytes and myocytes but there are limited studies in β‐cells. Work is needed to characterise the mechanism underpinning LC‐FFA uptake specifically in β‐cells. In conducting future research in β‐cells, studies should consider interspecies differences between candidate LC‐FFA transporters, the specificity of the transporter for LC‐FFA species, and the concentration of FFA that the transporter is exposed to due to potential saturable effects. Only upon determining this mechanism can we move towards effective treatments that prevent the potential deleterious effects of β‐cell lipotoxicity in the development of T2D.

## FUNDING INFORMATION

We gratefully acknowledge funding from the Medical Research Council (MR/T003391/1).

## CONFLICT OF INTEREST STATEMENT

The authors declare that they have no conflicts of interest regarding the publication of this article.

## Supporting information


**Table S1** Summary of included studies to identify candidate LC‐FFA transport proteins.
