# Supplementary material for: The role of candidate transport proteins in β‐cell long‐chain fatty acid uptake: Where are we now?
Source: Diabet Med. 2023 Sep 2;40(12):e15198. doi: 10.1111/dme.15198 (PMC10947460; doi:10.1111/dme.15198)
Supplement: Supplementary file 1 — Table S1 Summary of included studies to identify candidate LC‐FFA transport proteins. [file DME-40-0-s001.zip › Supplementary.docx]

**Identification of candidate LC-FFA transport proteins in the published literature**

We independently searched three electronic databases (Google Scholar, PubMed and Web of Science) using a set of reference keywords, obtaining 1588 articles. Following the Preferred Reporting Items for Systematic Reviews and Meta-Analysis (PRISMA) extension for scoping reviews framework (Tricco et al. 2018), references were screened for peer-reviewed experimental studies involving healthy mammalian cells/individuals, investigating LC-FFA candidate transport protein activity, resulting in 45 studies for close analysis. This approach gave us confidence that we included all relevant work that was of sufficiently high quality to warrant further investigation. The resulting list of candidate LC-FFA transport proteins (extracted from these 45 studies) is displayed in Table S1.

| **Table S1 Summary of included studies to identify candidate LC-FFA transport proteins** | | | | | | |
| --- | --- | --- | --- | --- | --- | --- |
| *Candidate transport protein* | *Ref.* | *Cell type* | *Model organism* | *Experimental conditions* | *LC-FFA transported* | *Was LC-FFA uptake facilitated* |
| CD36 | Jay et al. (2020) | Adipocyte | Rat | Chemical inhibition | *C18:1 | No |
| CD36 | Febbraio et al. (1999) | Adipocytes | Mouse | Knockout | C18:1 | Yes |
| CD36 | Hao et al. (2020) | Adipocytes (3T3-L1) | Mouse | Knockout | C18:1 | Yes |
| CD36 | Pohl et al. (2005) | Adipocytes (3T3-L1) | Mouse | Chemical inhibition | C18:1 | Yes |
| CD36 | Lobo et al. (2009) | Adipocytes (3T3-L1) | Mouse | Knockdown (shRNA) | **C16:0 | Yes |
| CD36 | Mitchell et al. (2011) | Brain microvessel endothelial cells (HBMEC) | Human | Knockdown (siRNA) | C18:1 and C16:0 | Yes |
| CD36 | Angin et al. (2012) | Cardiomyocytes | Rat | Chemical inhibition | C16:0 | Yes |
| CD36 | Steinbusch et al. (2010) | Cardiomyocytes | Rat | Chemical inhibition | C16:0 | Yes |
| CD36 | Luiken et al. (2001) | Cardiomyocytes | Rat | Chemical inhibition | C16:0 | Yes |
| CD36 | Carley & Kleinfeld (2011) | Cardiomyocytes | Mouse | Knockout | C18:1 | Yes (at 20nM, not at 100 or 200nM) |
| CD36 | Xu et al. (2013) | Embryonic kidney cells (HEK293T) | Human | Overexpressed | C18:1 | No |
| CD36 | Lynes et al. (2011) | Enterocytes | Mouse | Chemical inhibition | C16:0 | Yes (high fat diet)  No (normal fat diet) |
| CD36 | Schneider et al. (2014) | Epithelial kidney cells (MDCK) | Dog | Overexpressed | C18:1 | Yes |
| CD36 | Ehehalt et al. (2008) | Fibroblast-like cell line (COS) | Grivet | Overexpressed | C18:1 | Yes |
| CD36 | Ibrahimi et al. (1996) | Fibroblasts (Obl7PY) | Mouse | Overexpressed | C16:0 and C18:1 | Yes |
| CD36 | Bonen et al. (2000) | Giant sarcolemmal vesicles | Rat | Chemical inhibition | C16:0 | Yes |
| CD36 | Luiken et al. (1999) | Giant sarcolemmal vesicles (heart and muscle) | Rat | Chemical inhibition | C16:0 | Yes |
| CD36 | Chabowski et al. (2013) | Hepatocytes | Rat | Chemical inhibition | C16:0 and C18:1 | Yes |
| CD36 | Chabowski et al. (2005) | Myocytes | Rat | Chemical inhibition | C16:0 | Yes |
| CD36 | Garcia-Martinez et al. (2005) | Myocytes | Human | Overexpressed | C16:0 and C18:1 | Yes |
| CD36 | Benninghoff et al. (2020) | Myocytes | Mouse | Knockdown (siRNAs) | C16:0 and C18:1 | Yes |
| CD36 | Luiken et al. (2002) | Myocytes | Rat | Chemical inhibition | C16:0 | Yes |
| CD36 | Guthmann et al. (1999) | Pneumocytes (type II) | Rat | Chemical inhibition | C16:0 | Yes |
| CD36 | Hames et al. (2014) | Adipocytes, Myocytes (skeletal muscle), cardiomyocytes, hepatocytes | Human | In vivo | C16:0 | Yes (adipocytes, myocytes and cardiomyocytes)  No (hepatocytes) |
| CD36 | Salameh et al. (2016) | Adipocytes (3T3-L1) | Mouse | Chemical inhibition | C16:0 | Yes |
| CD36 | Momken et al. (2017) | Cardiomyocytes, skeletal muscle (giant sarcolemmal vesicles) | Mouse | Chemical inhibition (cardiomyocytes), Knockout (skeletal muscle) | C16:0 | Yes |
| CD36 | Wang et al. (2019) | Embryonic kidney cells (HEK293T), preadipocytes (3T3-L1) | Human | Knockdown (shRNA) (HEK293), Overexpressed (3T3-L1) | C18:1 | Yes |
| CAV1 | Hao et al. (2020) | Adipocytes (3T3-L1) | Mouse | Knockout | C18:1 | Yes |
| CAV1 | Meshulam et al. (2006) | Embryonic kidney cells (HEK293T) | Human | Overexpressed | C16:0 and C18:1 | Yes |
| CAV1 | Schneider et al. (2014) | Epithelial kidney cells (MDCK) | Dog | Knockdown (RNAi) | C18:1 | No |
| CAV1 | Siddiqi et al. (2013) | Intestinal brush border | Rat and Mouse | Knockout | C18:1 | Yes |
| CAV3 | Pohl et al. (2004) | Adipocytes (3T3-L1), Fibroblasts (3T3-L1) | Mouse | Chemical inhibition | C18:1 and C18:0 | Yes (adipocytes)  No (fibroblasts) |
| FATP1 | Lobo et al. (2007) | Adipocytes (3T3-L1) | Mouse | Knockdown (shRNA) | C16:0 and C18:1 | Yes |
| FATP1 | Gargiulo et al. (1999) | Adipocytes (3T3-L1) | Mouse | Overexpressed | C18:1 | Yes |
| FATP1 | Wiczer et al. (2009) | Adipocytes (3T3-L1) | Mouse | Knockdown (shRNA) | C16:0 | Yes |
| FATP1 | Schaffer & Lodish (1994) | Adipocytes (3T3-L1) | Mouse | Overexpressed | C16:0 and C18:1 | Yes |
| FATP1 | Mitchell et al. (2011) | Brain microvessel endothelial cells (HBMEC) | Human | Knockdown (siRNAs) | C16:0 and C18:1 | Yes |
| FATP1 | Holloway et al. (2011) | Giant sarcolemmal vesicles | Rat | Overexpressed | C16:0 | Yes |
| FATP1 | Garcia-Martinez et al. (2005) | Myocytes | Human | Overexpressed | C16:0 and C18:1 | Yes |
| FATP1 | Wu et al. (2006) | Adipocytes (3T3-L1), myocytes | Mouse | Knockout | C18:1 | No (unstimulated cells), Yes (insulin stimulated cells) |
| FATP4 | Lobo et al. (2007) | Adipocytes (3T3-L1) | Mouse | Knockdown (shRNA) | C16:0 and C18:1 | No |
| FATP4 | Mitchell et al. (2011) | Brain microvessel endothelial cells (HBMEC) | Human | Knockdown (siRNAs) | C16:0 and C18:1 | No |
| FATP4 | Benninghoff et al. (2020) | Myocytes | Mouse | Knockdown (siRNAs) | C16:0 and C18:1 | Yes |
| FATP4 | Stahl et al. (1999) | Embryonic kidney cells (HEK293T), enterocytes | Human (HEK293), Mouse (enterocytes) | Overexpressed (HEK293), Chemical inhibition (enterocytes) | C16:0 and C18:1 | Yes |
| FATP6 | Gimeno et al. (2003) | Fibroblast-like cell line (COS) | Monkey | Overexpressed | C16:0 and C18:1 | Yes |
| ACSL1 | Gargiulo et al. (1999) | Adipocytes (3T3-L1) | Mouse | Overexpressed | C18:1 | Yes |
| ACSL1 | Lobo et al. (2009) | Adipocytes (3T3-L1) | Mouse | Knockdown (shRNA) | C16:0 | No |
| FABP1 | Zhou et al. (1992) | Adipocytes (3T3-L1) | Mouse | Chemical inhibition | C18:1 | Yes |
| FABP1 | Newberry et al. (2003) | Hepatocytes | Mouse | Knockout | C18:1 | Yes (in the fasted state)  No (in the fed state) |
| FABP5 | Mitchell et al. (2011) | Brain microvessel endothelial cells (HBMEC) | Human | Knockdown (siRNAs) | C16:0 and C18:1 | Yes |
| FABPpm | Luiken et al. (1999) | Giant sarcolemmal vesicles (heart and muscle) | Rat | Chemical inhibition | C16:0 | Yes |
| FABPpm | Turcotte et al. (2000) | Giant sarcolemmal vesicles | Rat | Chemical inhibition | C16:0 | Yes |
| FABPpm | Sorrentino et al. (1988) | Myocytes | Rat | Chemical inhibition | C18:1 | Yes |
| FABP8 | Stremmel (1988) | Jejunal muscle cells | Rat | Chemical inhibition | C16:0 and C18:1 | Yes |
| TBC1D1 | Miklosz et al. (2021) | Adipose-derived mesenchymal stem cells (ADMSCs) | Human | Knockdown (siRNA) | C16:0 | No |
| TBC1D1 | Benninghoff et al. (2020) | Myocytes | Mouse | Knockdown (siRNA) | C16:0 and C18:1 | Yes |
| TBC1D4 | Miklosz et al. (2021) | Adipose-derived mesenchymal stem cells (ADMSCs) | Human | Knockdown (siRNA) | C16:0 | Yes |
| TBC1D4 | Benninghoff et al. (2020) | Myocytes | Mouse | Knockdown (siRNA) | C16:0 and C18:1 | Yes |
| TM4SF5 | Park et al. (2021) | Hepatocytes | Mouse | Knockdown (siRNA), chemical inhibition, overexpressed | C16:0 | Yes |
| Rab8a | Benninghoff et al. (2020) | Myocytes | Mouse | Knockdown (siRNAs) | C16:0 and C18:1 | Yes |
| Rab8b | Benninghoff et al. (2020) | Myocytes | Mouse | Knockdown (siRNAs) | C16:0 and C18:1 | Yes |
| Rab10 | Benninghoff et al. (2020) | Myocytes | Mouse | Knockdown (siRNAs) | C16:0 and C18:1 | Yes |
| Rab14 | Benninghoff et al. (2020) | Myocytes | Mouse | Knockdown (siRNAs) | C16:0 and C18:1 | Yes |
| Rab28 | Benninghoff et al. (2020) | Myocytes | Mouse | Knockdown (siRNAs) | C16:0 and C18:1 | No |
| PHB | Salameh et al. (2016) | Adipocytes (3T3-L1), endothelial cells derived from brain tissue (bEnd.3) | Mouse | Knockdown (3T3-L1), overexpression (siRNA) | C16:0 | Yes |
| ANX2 | Salameh et al. (2016) | Adipocytes (3T3-L1) | Mouse | Knockdown (3T3-L1) | C16:0 | Yes |

**C18:1 oleic acid, **C16:0: palmitate,*

**Reference list of literature review studies as shown in Table S1**

1. Angin Y, Steinbusch LK, Simons PJ, et al. CD36 inhibition prevents lipid accumulation and contractile dysfunction in rat cardiomyocytes. *Biochem J*. 2012;448(1):43-53. doi:10.1042/BJ20120060.
2. Benninghoff T, Espelage L, Eickelschulte S, et al. The RabGAPs TBC1D1 and TBC1D4 Control Uptake of Long-Chain Fatty Acids Into Skeletal Muscle via Fatty Acid Transporter SLC27A4/FATP4. *Diabetes*. 2020;69(11):2281-2293. doi:10.2337/db20-0180.
3. Bonen A, Luiken JJ, Arumugam Y, Glatz JF, Tandon NN. Acute regulation of fatty acid uptake involves the cellular redistribution of fatty acid translocase. *J Biol Chem*. 2000;275(19):14501-14508. doi:10.1074/jbc.275.19.14501
4. Carley AN, Kleinfeld AM. Fatty acid (FFA) transport in cardiomyocytes revealed by imaging unbound FFA is mediated by an FFA pump modulated by the CD36 protein. *J Biol Chem*. 2011;286(6):4589-4597. doi:10.1074/jbc.M110.182162.
5. Chabowski A, Żendzian-Piotrowska M, Konstantynowicz K, et al. Fatty acid transporters involved in the palmitate and oleate induced insulin resistance in primary rat hepatocytes. *Acta Physiol (Oxf)*. 2013;207(2):346-357. doi:10.1111/apha.12022.
6. Chabowski A, Coort SL, Calles-Escandon J, et al. The subcellular compartmentation of fatty acid transporters is regulated differently by insulin and by AICAR. *FEBS Lett*. 2005;579(11):2428-2432. doi:10.1016/j.febslet.2004.11.118.
7. Ehehalt R, Sparla R, Kulaksiz H, Herrmann T, Füllekrug J, Stremmel W. Uptake of long chain fatty acids is regulated by dynamic interaction of FAT/CD36 with cholesterol/sphingolipid enriched microdomains (lipid rafts). *BMC Cell Biol*. 2008;9:45. doi:10.1186/1471-2121-9-45.
8. Febbraio M, Abumrad NA, Hajjar DP, et al. A null mutation in murine CD36 reveals an important role in fatty acid and lipoprotein metabolism. *J Biol Chem*. 1999;274(27):19055-19062. doi:10.1074/jbc.274.27.19055.
9. García-Martínez C, Marotta M, Moore-Carrasco R, et al. Impact on fatty acid metabolism and differential localization of FATP1 and FAT/CD36 proteins delivered in cultured human muscle cells. *Am J Physiol Cell Physiol*. 2005;288(6):C1264-C1272. doi:10.1152/ajpcell.00271.2004.
10. Gargiulo CE, Stuhlsatz-Krouper SM, Schaffer JE. Localization of adipocyte long-chain fatty acyl-CoA synthetase at the plasma membrane. *J Lipid Res*. 1999;40(5):881-892.
11. Gimeno RE, Ortegon AM, Patel S, et al. Characterization of a heart-specific fatty acid transport protein. *J Biol Chem*. 2003;278(18):16039-16044. doi:10.1074/jbc.M211412200.
12. Guthmann F, Haupt R, Looman AC, Spener F, Rüstow B. Fatty acid translocase/CD36 mediates the uptake of palmitate by type II pneumocytes. *Am J Physiol*. 1999;277(1):L191-L196. doi:10.1152/ajplung.1999.277.1.L191.
13. Hames KC, Vella A, Kemp BJ, Jensen MD. Free fatty acid uptake in humans with CD36 deficiency. *Diabetes*. 2014;63(11):3606-3614. doi:10.2337/db14-0369.
14. Hao JW, Wang J, Guo H, et al. CD36 facilitates fatty acid uptake by dynamic palmitoylation-regulated endocytosis. *Nat Commun*. 2020;11(1):4765. Published 2020 Sep 21. doi:10.1038/s41467-020-18565-8.
15. Holloway GP, Chou CJ, Lally J, et al. Increasing skeletal muscle fatty acid transport protein 1 (FATP1) targets fatty acids to oxidation and does not predispose mice to diet-induced insulin resistance. *Diabetologia*. 2011;54(6):1457-1467. doi:10.1007/s00125-011-2114-8.
16. Ibrahimi A, Sfeir Z, Magharaie H, Amri EZ, Grimaldi P, Abumrad NA. Expression of the CD36 homolog (FAT) in fibroblast cells: effects on fatty acid transport. *Proc Natl Acad Sci U S A*. 1996;93(7):2646-2651. doi:10.1073/pnas.93.7.2646.
17. Jay AG, Simard JR, Huang N, Hamilton JA. SSO and other putative inhibitors of FA transport across membranes by CD36 disrupt intracellular metabolism, but do not affect FA translocation. *J Lipid Res*. 2020;61(5):790-807. doi:10.1194/jlr.RA120000648.
18. Lobo S, Wiczer BM, Bernlohr DA. Functional analysis of long-chain acyl-CoA synthetase 1 in 3T3-L1 adipocytes. *J Biol Chem*. 2009;284(27):18347-18356. doi:10.1074/jbc.M109.017244.
19. Lobo S, Wiczer BM, Smith AJ, Hall AM, Bernlohr DA. Fatty acid metabolism in adipocytes: functional analysis of fatty acid transport proteins 1 and 4. *J Lipid Res*. 2007;48(3):609-620. doi:10.1194/jlr.M600441-JLR200.
20. Luiken JJ, Koonen DP, Willems J, et al. Insulin stimulates long-chain fatty acid utilization by rat cardiac myocytes through cellular redistribution of FAT/CD36. *Diabetes*. 2002;51(10):3113-3119. doi:10.2337/diabetes.51.10.3113.
21. Luiken JJ, Willems J, van der Vusse GJ, Glatz JF. Electrostimulation enhances FAT/CD36-mediated long-chain fatty acid uptake by isolated rat cardiac myocytes. *Am J Physiol Endocrinol Metab*. 2001;281(4):E704-E712. doi:10.1152/ajpendo.2001.281.4.E704.
22. Luiken JJ, Turcotte LP, Bonen A. Protein-mediated palmitate uptake and expression of fatty acid transport proteins in heart giant vesicles. *J Lipid Res*. 1999;40(6):1007-1016.
23. Lynes M, Narisawa S, Millán JL, Widmaier EP. Interactions between CD36 and global intestinal alkaline phosphatase in mouse small intestine and effects of high-fat diet. *Am J Physiol Regul Integr Comp Physiol*. 2011;301(6):R1738-R1747. doi:10.1152/ajpregu.00235.2011.
24. Meshulam T, Simard JR, Wharton J, Hamilton JA, Pilch PF. Role of caveolin-1 and cholesterol in transmembrane fatty acid movement. *Biochemistry*. 2006;45(9):2882-2893. doi:10.1021/bi051999b.
25. Mikłosz A, Łukaszuk B, Supruniuk E, et al. Does TBC1D4 (AS160) or TBC1D1 Deficiency Affect the Expression of Fatty Acid Handling Proteins in the Adipocytes Differentiated from Human Adipose-Derived Mesenchymal Stem Cells (ADMSCs) Obtained from Subcutaneous and Visceral Fat Depots?. *Cells*. 2021;10(6):1515. doi:10.3390/cells10061515.
26. Mitchell RW, On NH, Del Bigio MR, Miller DW, Hatch GM. Fatty acid transport protein expression in human brain and potential role in fatty acid transport across human brain microvessel endothelial cells. *J Neurochem*. 2011;117(4):735-746. doi:10.1111/j.1471-4159.2011.07245.x.
27. Momken I, Chabowski A, Dirkx E, et al. A new leptin-mediated mechanism for stimulating fatty acid oxidation: a pivotal role for sarcolemmal FAT/CD36. *Biochem J*. 2017;474(1):149-162. doi:10.1042/BCJ20160804.
28. Newberry EP, Xie Y, Kennedy S, et al. Decreased hepatic triglyceride accumulation and altered fatty acid uptake in mice with deletion of the liver fatty acid-binding protein gene. *J Biol Chem*. 2003;278(51):51664-51672. doi:10.1074/jbc.M309377200.
29. Park D, Kim E, Lee H, Shin EA, Lee H, Lee JW. Tetraspanin TM4SF5 in hepatocytes negatively modulates SLC27A transporters during acute fatty acid supply. *Arch Biochem Biophys*. 2021;710:109004. doi:10.1016/j.abb.2021.109004.
30. Pohl J, Ring A, Ehehalt R, et al. Long-chain fatty acid uptake into adipocytes depends on lipid raft function. *Biochemistry*. 2004;43(14):4179-4187. doi:10.1021/bi035743m.
31. Pohl J, Ring A, Korkmaz U, Ehehalt R, Stremmel W. FAT/CD36-mediated long-chain fatty acid uptake in adipocytes requires plasma membrane rafts. *Mol Biol Cell*. 2005;16(1):24-31. doi:10.1091/mbc.e04-07-0616.
32. Salameh A, Daquinag AC, Staquicini DI, et al. Prohibitin/annexin 2 interaction regulates fatty acid transport in adipose tissue. *JCI Insight*. 2016;1(10):e86351. doi:10.1172/jci.insight.86351.
33. Schaffer JE, Lodish HF. Expression cloning and characterization of a novel adipocyte long chain fatty acid transport protein. *Cell*. 1994;79(3):427-436. doi:10.1016/0092-8674(94)90252-6.
34. Schneider H, Staudacher S, Poppelreuther M, Stremmel W, Ehehalt R, Füllekrug J. Protein mediated fatty acid uptake: synergy between CD36/FAT-facilitated transport and acyl-CoA synthetase-driven metabolism. *Arch Biochem Biophys*. 2014;546:8-18. doi:10.1016/j.abb.2014.01.025.
35. Siddiqi S, Sheth A, Patel F, Barnes M, Mansbach CM 2nd. Intestinal caveolin-1 is important for dietary fatty acid absorption. *Biochim Biophys Acta*. 2013;1831(8):1311-1321. doi:10.1016/j.bbalip.2013.05.001.
36. Sorrentino D, Stump D, Potter BJ, et al. Oleate uptake by cardiac myocytes is carrier mediated and involves a 40-kD plasma membrane fatty acid binding protein similar to that in liver, adipose tissue, and gut. *J Clin Invest*. 1988;82(3):928-935. doi:10.1172/JCI113700.
37. Stahl A, Hirsch DJ, Gimeno RE, et al. Identification of the major intestinal fatty acid transport protein. *Mol Cell*. 1999;4(3):299-308. doi:10.1016/s1097-2765(00)80332-9.
38. Steinbusch LK, Wijnen W, Schwenk RW, et al. Differential regulation of cardiac glucose and fatty acid uptake by endosomal pH and actin filaments. *Am J Physiol Cell Physiol*. 2010;298(6):C1549-C1559. doi:10.1152/ajpcell.00334.2009.
39. Stremmel W. Uptake of fatty acids by jejunal mucosal cells is mediated by a fatty acid binding membrane protein. *J Clin Invest*. 1988;82(6):2001-2010. doi:10.1172/JCI113820.
40. Turcotte LP, Swenberger JR, Tucker MZ, et al. Muscle palmitate uptake and binding are saturable and inhibited by antibodies to FABP(PM). *Mol Cell Biochem*. 2000;210(1-2):53-63. doi:10.1023/a:1007046929776.
41. Wang J, Hao JW, Wang X, et al. DHHC4 and DHHC5 Facilitate Fatty Acid Uptake by Palmitoylating and Targeting CD36 to the Plasma Membrane. *Cell Rep*. 2019;26(1):209-221.e5. doi:10.1016/j.celrep.2018.12.022.
42. Wiczer BM, Bernlohr DA. A novel role for fatty acid transport protein 1 in the regulation of tricarboxylic acid cycle and mitochondrial function in 3T3-L1 adipocytes. *J Lipid Res*. 2009;50(12):2502-2513. doi:10.1194/jlr.M900218-JLR200.
43. Wu Q, Ortegon AM, Tsang B, Doege H, Feingold KR, Stahl A. FATP1 is an insulin-sensitive fatty acid transporter involved in diet-induced obesity. *Mol Cell Biol*. 2006;26(9):3455-3467. doi:10.1128/MCB.26.9.3455-3467.2006.
44. Xu S, Jay A, Brunaldi K, Huang N, Hamilton JA. CD36 enhances fatty acid uptake by increasing the rate of intracellular esterification but not transport across the plasma membrane. *Biochemistry*. 2013;52(41):7254-7261. doi:10.1021/bi400914c.
45. Zhou SL, Stump D, Sorrentino D, Potter BJ, Berk PD. Adipocyte differentiation of 3T3-L1 cells involves augmented expression of a 43-kDa plasma membrane fatty acid-binding protein. *J Biol Chem*. 1992;267(20):14456-14461.
