# Supplementary material for: The role of candidate transport proteins in β‐cell long‐chain fatty acid uptake: Where are we now?
Source: Diabet Med. 2023 Sep 2;40(12):e15198. doi: 10.1111/dme.15198 (PMC10947460; doi:10.1111/dme.15198)
Supplement: Supplementary file 1 — Table S1 Summary of included studies to identify candidate LC‐FFA transport proteins. [file DME-40-0-s001.zip › Supporting_information.docx]

**List of abbreviations**

Endoplasmic reticulum ER

Fatty acid binding protein FABP

Fatty acid translocase FAT

Fatty acid transport protein FATP

Free fatty acids FFA

Free fatty acid receptor FFAR

G protein-coupled receptor GPCR

Glucose-stimulated insulin secretion GSIS

Long-chain monounsaturated fatty acids LC-MUFA

Long-chain free fatty acids LC-FFA

Long-chain saturated fatty acids LC-SFA

Peroxisome proliferator-activated receptors PPAR

Type 2 diabetes T2D
